# Genetic Analysis of High Bone Mass Cases from the BARCOS Cohort of Spanish Postmenopausal Women

**DOI:** 10.1371/journal.pone.0094607

**Published:** 2014-04-15

**Authors:** Patricia Sarrión, Leonardo Mellibovsky, Roser Urreizti, Sergi Civit, Neus Cols, Natàlia García-Giralt, Guy Yoskovitz, Alvaro Aranguren, Jorge Malouf, Silvana Di Gregorio, Luís Del Río, Roberto Güerri, Xavier Nogués, Adolfo Díez-Pérez, Daniel Grinberg, Susana Balcells

**Affiliations:** 1 Departament de Genètica, Universitat de Barcelona, IBUB, Barcelona, Spain; 2 Centro de Investigación Biomédica en Red de Enfermedades Raras (CIBERER), Instituto de Salud Carlos III, Barcelona, Spain; 3 Institut de Biomedicina Universitat de Barcelona (IBUB), Barcelona, Spain; 4 Unitat de Recerca en Fisiologia Òssia i Articular (URFOA), Institut Municipal d'Investigacions Mèdiques (IMIM), Hospital del Mar, Barcelona, Spain; 5 Red Tematica de Investigación Cooperativa en Envejecimiento y Fragilidad (RETICEF), Instituto de Salud Carlos III, Barcelona, Spain; 6 Departament d'Estadística, Universitat de Barcelona, Barcelona, Spain; 7 Hospital de la Santa Creu i Sant Pau, Barcelona, Spain; 8 CETIR Medical Imaging Centre, Barcelona, Spain; Van Andel Institute, United States of America

## Abstract

The aims of the study were to establish the prevalence of high bone mass (HBM) in a cohort of Spanish postmenopausal women (BARCOS) and to assess the contribution of *LRP5* and *DKK1* mutations and of common bone mineral density (BMD) variants to a HBM phenotype. Furthermore, we describe the expression of several osteoblast-specific and Wnt-pathway genes in primary osteoblasts from two HBM cases. A 0.6% of individuals (10/1600) displayed Z-scores in the HBM range (sum Z-score >4). While no mutation in the relevant exons of *LRP5* was detected, a rare missense change in *DKK1* was found (p.Y74F), which cosegregated with the phenotype in a small pedigree. Fifty-five BMD SNPs from Estrada *et al.* [NatGenet 44:491-501,2012] were genotyped in the HBM cases to obtain risk scores for each individual. In this small group of samples, Z-scores were found inversely related to risk scores, suggestive of a polygenic etiology. There was a single exception, which may be explained by a rare penetrant genetic variant, counterbalancing the additive effect of the risk alleles. The expression analysis in primary osteoblasts from two HBM cases and five controls suggested that *IL6R*, *DLX3*, *TWIST1* and *PPARG* are negatively related to Z-score. One HBM case presented with high levels of *RUNX2*, while the other displayed very low *SOX6*. In conclusion, we provide evidence of lack of *LRP5* mutations and of a putative HBM-causing mutation in *DKK1*. Additionally, we present SNP genotyping and expression results that suggest additive effects of several genes for HBM.

## Introduction

Osteoporosis has a complex genetic background. Bone mineral density (BMD) is a highly heritable intermediate phenotype that correlates well with fracture risk [1]–[6]. BMD is distributed as a Gaussian curve in the general population, with two small groups having extremely low or extremely high BMD values at both ends. These individuals with extreme phenotypes may bear infrequent and highly penetrant alleles at a few specific loci. Alternatively, the extreme phenotypes may depend on the presence of a high number of common variants with low penetrance and additive effects.

A few individuals with high bone mass (HBM, MIM#601884), as defined by a sum Z-score >4 (total lumbar spine Z-score + total femoral neck Z-score), have been reported to bear highly penetrant missense alleles at the low-density lipoprotein receptor-related protein 5 (*LRP5,* MIM#603506) locus that are transmitted in an autosomal dominant way. More than 10 years ago, two different groups found that *LRP5* regulated bone mass [7], [8]. While inactivating mutations in *LRP5* were shown to cause osteoporosis-pseudoglioma syndrome [7], gain-of-function mutations caused a high bone mass (HBM) phenotype [8]. This phenotype has been associated with the *LRP5*–G171V mutation in two independent pedigrees [8], [9]. Six additional missense mutations (D111Y, G171R, A214T, A214V, A242T and T253I), all in the first β-propeller domain of *LRP5*, were identified in patients who also showed an increased bone density [10]. The affected individuals had elevated bone synthesis assessed by serum markers, but normal bone resorption, bone architecture and serum calcium, phosphate, PTH and vitamin D levels [8], [9]. Significant phenotypic heterogeneity was reported, and some affected family members also had a torus palatinus.

LRP5 acts as a co-receptor with members of the Frizzled family to activate the canonical Wnt/β-catenin signalling pathway, which is crucial for bone formation [11]. This pathway is activated by the binding of the appropriate Wnt protein to LRP5 and is blocked by the binding of inhibitors such as Dickkopf-related protein 1 (encoded by *DKK1*, MIM#605189) and Sclerostin (encoded by *SOST*). The HBM-causing mutation prevents the binding of these two inhibitors. Mutations in *SOST* are the cause of van Buchem disease [12] and sclerostosis [13], two pathologies with an abnormally high bone density. On the other hand, *Dkk1* ^+/−^ mice showed a marked increase in bone mass [14].

The prevalence of HBM in the general population has been estimated as 0.2–1% [8], [15], [16], but the genetic architecture of this extreme phenotype remains poorly understood. However, recent genome-wide association (GWA) analyses and meta-analyses have established a number of genomic loci that explain differences in BMD across the general population. In particular, Estrada et al. [17] identified 56 such genomic loci and showed how they can be used to calculate risk scores to predict BMD.

In order to explore the genetic constitution of a high bone mass phenotype, our aims were, first, to establish the prevalence of HBM in the BARCOS (BARCelona OSteoporosis) cohort of postmenopausal Spanish women; second, to determine whether any of the HMB cases carried *LRP5* or *DKK1* mutations that could explain the phenotype; and third, to assess whether the HBM cases were carriers of a low number of risk alleles of 55 autosomal GWA-identified BMD loci. Also, we took advantage of the availability of primary osteoblasts from two HBM cases to characterize the osteoblast RNA in terms of osteoblast-specific and/or Wnt-pathway genes by comparison with osteoblast RNA from donors with normal or low BMD.

## Materials and Methods

### Ethics Statement

Both the Bioethics Committee of Universitat de Barcelona and the Clinical Research Ethics Committee of Parc de Salud MAR have emitted a favourable bioethical statement regarding the present research. Specifically, the protocol for obtention of peripheral blood from the BARCOS cohort women and the protocol for the obtention of primary osteoblasts from bone specimens extracted from knee samples otherwise discarded at the time of orthopaedic surgery, were approved by both committees. Written informed consents were obtained from the participants in both instances.

### Study Cohort

The study population (listed in Table 1) included the HBM cases in the BARCOS cohort (n = 10 unrelated cases). This cohort of postmenopausal women from the Barcelona area has been described elsewhere [18], [19]. At present time, it includes DXA values for 1600 women and DNA samples for 1001 of them. Six additional unrelated female HBM cases were recruited from 3 hospitals of Barcelona, Hospital de Sant Pau (1 case), CETIR (a private medical services centre specializing in nuclear medicine and other imaging modalities, 4 cases), and Hospital de L'Esperança (1 case). Some relatives of particular cases were also studied. Blood samples and written informed consent were obtained in accordance with the regulations of the Clinical Research Ethics Committee of Parc de Salud MAR. A total of 1600 dual-energy X-ray absorptiometry scans (DXA; QDR 4500 SL; Hologic, Waltham, MA, USA) of the women from this cohort were analysed in order to pinpoint those HBM cases in which the sum Z-score (hip plus lumbar spine) was equal to or greater than four [8]. All DXA measurements were performed prior to any treatment that could increase bone mass. Pathologic phenotypes such as osteopetrosis or any other sclerosing bone disorders were ruled out based on the absence of radiologic alterations in the skull and long bones, the absence of any fragility fracture and the absence of any underlying disease. For individuals HBM1, HBM8, and HBM11, no DNA sample was available.

**Table 1:** Z-score values, age and cohort of the participant women.

Case	Sum Z-score	LS Z-score	HIP Z-score	Age	Cohort
HBM1^1^^,^ 2	6.1	3.4	2.7	51	BARCOS
HBM2	4.6	3.0	1.6	57	BARCOS
HBM3	4.9	2.5	2.4	55	BARCOS
HBM4	4.5	2.5	2.0	62	BARCOS
HBM5	4.5	2.4	2.1	66	BARCOS
HBM6	5.1	2.6	2.5	52	BARCOS
HBM7	4.6	2.5	2.1	61	BARCOS
HBM8^2^	7.9	4.0	3.9	55	BARCOS
HBM9	7.0	4.6	2.4	66	BARCOS
HBM10	5.1	2.8	2.3	75	BARCOS
HBM11^2^	6.8	3.8	3.0	55	HSANTPAU
HBM12	6.4	3.8	2.6	59	CETIR
HBM13	5.2	2.6	2.6	67	CETIR
HBM14	6.0	3.7	2.3	64	CETIR
HBM15	4.5	2.4	2.1	54	CETIR
HBM16	5.3	3.6	1.7	77	HESP

1 Deceased during the course of the study.

2 No DNA sample available.

### Sequencing of *LRP5* and *DKK1*

The genomic DNA of n = 13 HBM cases (Table 2) was isolated from peripheral blood leukocytes using conventional methods. In all probands, *LRP5* exons 2 to 4 (encoding the first beta-propeller and harbouring all HBM-related mutations described so far) and exons 9 to 16 (encoding the third and fourth beta-propellers involved in binding to DKK1), and their intronic flanking regions, and the four exons and flanking regions of *DKK1* were amplified and sequenced using specific primers. Mutation screening was performed by direct sequencing using the BigDye v3.1 kit (Applied Biosystems, Foster City, CA, USA) and the ABI PRISM 3730 DNA Analyzer (Applied Biosystems). Primers and PCR conditions are listed in Table S1. Nomenclature for DNA variants followed these reference sequences: NM_002335.2 for *LRP5* and NM_012242.2 for *DKK1*.

**Table 2:** Genotypes at *LRP5* and *DKK1*.

	LRP5 exonic SNPs				DKK1 exonic changes	
Case	p.V667M^1^	p.A1330V^1^	p.V1119V^1^	Others^1^	p.A106A^1^	Variant
HBM2	-	-	Het.	p.E644E	Het.	-
HBM3	-	-	Het.	p.N705N	Homo.	-
HBM4	-	Het.	Het.	p.N740N	Het.	-
HBM5	-	-	Het.	p.E644E	-	-
HBM6	-	-	-	-	Homo.	-
HBM7	-	-	Het.	p.N740N	-	-
HBM9	-	Het.	Het.	-	Het.	-
HBM10	-	-	Het.	-	-	-
HBM12	-	-	Het.	p.E644E	Het.	-
HBM13	Het.	Het.	Het.	p.N740N	Het.	-
HBM14	-	Het.	Het.	p.N740N	Het.	-
HBM15	-	-	-	-	-	**p.Y74F** 2
HBM16	-	-	-	p.E644E	-	-

1 Corresponding reference sequences, rs-numbers and MAFs are: LRP5 (NM_002335.2): p.E644E (rs2277268. 0.06); p.V667 (rs4988321. 0.03); p.N705N (rs145456776. <0.01); p.N740N (rs2306862. 0.15); p.V1119V (rs556442. 0.28); p.A1330V (rs3736228. 0.13). DKK1 (NM_012242.2): p.A106A (rs2241529. 0.46). All LRP5 variants listed under “Others”. as well as the DKK1 p.Y74F were found in heterozygous state.

2 A novel missense change is indicated in bold. Het.: heterozygous for the variant; Homo.: homozygous for the minor allele; NA: not available; -: homozygous for the reference allele.

### SNP Selection and Genotyping

Out of the 64 SNPs identified by Estrada et al. [17], we chose 55, one for each autosomal locus (listed in Table S2). Genotyping of the 13 available HBM cases was carried out with a KASPar v4.0 genotyping system at the Kbioscience facilities (KBioscience, Herts, UK) using the Kraken allele-calling algorithm [20]. The genotypes of 1001 BARCOS participants for the same SNPs were already available, since this cohort was included in the replication phase of the study by Estrada et al. [17]. One of the SNPs (rs3790160) gave conflicting results and was eliminated from the analyses. Quality control was carried out by resequencing 6.28% of the samples. The readings showed full concordance between the two techniques.

### Genetic Risk Allele Analysis

Fifty-five SNPs previously described to be associated with BMD at GWA significance [17] were genotyped in the 13 HBM cases and the genetic risk score for each individual was then calculated by taking into account both the number of risk alleles and the relative effect of each SNP on BMD, as carried out by Estrada et al. [17]. This calculation was also performed for 1001 individuals in the BARCOS cohort with available SNP genotype and LS-BMD data. Briefly, the genotype of each SNP was transformed into a risk score by taking into account the effects estimated by the authors and listed in their Supplementary Table 9. The effect size (beta parameter or slope) is the BMD decrease due to the presence of one copy of the risk allele. The scores for homozygotes for the risk allele were 2x the effect size; scores for heterozygotes were 1x the effect size; and the scores for homozygotes for the alternative allele were zero. For each individual, the risk scores of all SNPs were summed up to obtain a global risk score, which was then normalized by dividing it by the mean effect size of BARCOS. Normalized global risk scores were sorted into five bins, as described in Estrada et al. [17]. Missing genotypes within BARCOS cohort individuals and also within HBM probands were solved by replacing them by the mean of the corresponding SNP scores in BARCOS. This strategy would attenuate the variance of the overall group [21].

### Primary Osteoblast Isolation and Cell Culture

Primary osteoblast (hOB) cells of postmenopausal women were available from bone specimens extracted from knee samples that would otherwise have been discarded at the time of artroplasty. Both informed consent and BMD values were obtained from the donors. Two of them were patients HBM10 and HBM16. Five female donors with sum Z-score values (and ages) of 2.4 (85 y), 1.2 (74 y), 0.4 (79 y), −0.7 (70 y) and −2.2 (61 y), were used as controls. The bone tissue used was obtained from a region at least 2 cm apart from the subchondral and the osteochondral plates far from the described 6 mm layer of trabecular bone below the above mentioned plates [22]. With this we aimed to minimize the potential issues of using osteoblastic cells located close to the damaged joint (given the known relationship and mutual influences between the inflamed cartilage and adjacent bone cells) and the alterations in phenotype and gene expression of the osteoblasts in this zone [23], [24]. The hOB cells were obtained, as described previously [25], [26]. Briefly, the trabecular bone was separated and cut into small fragments, washed in phosphate buffered solution (PBS) to remove non-adherent cells, and placed on a petri dish. Samples were incubated in Dulbecco's Modified Eagle Medium (DMEM; Gibco; Invitrogen, Paisley, UK), supplemented with sodium pyruvate (1 mM), L-glutamine (1 mM), 1% penicillin/streptomycin, 10% fetal calf serum (FCS), 0.4% fungizone and 1% ascorbic acid. This allowed osteoblastic precursor cells to migrate from the fragments and proliferate. After confluence, cells were trypsinized and cultured in the same medium. When sub-confluence was reached again, the medium was aspirated and fresh medium with 10% serum or 0.1% bovine serum albumin was added. Forty-eight hours later, the medium was aspirated, cells were rinsed with phosphate-buffered saline, and the RNA was extracted. Standard histochemistry or quantitative PCR tests to measure alkaline phosphatase or osteocalcin expression, respectively, were used to confirm the osteoblastic nature of the cells, as described in [27].

### RNA Extraction, cDNA Synthesis and Real-time PCR

Total cell RNA was extracted using the High Pure RNA Isolation Kit (Roche Diagnostics, Mannheim, Germany) in accordance with the manufacturer's instructions. Two micrograms of total RNA were reverse transcribed using random primers of the High Capacity cDNA RT Kit (Applied Biosystems) in accordance with the manufacturer's instructions. The real-time PCR (qPCR) reactions were performed in a final volume of 10 µl using 20 ng of each cDNA, which was used as template for each well in the RealTime ready Custom Panel 384 (Roche Diagnostics). This custom panel included 88 genes selected by us, taking into account, among other sources, the sites recently highlighted by GWA analyses, in particular those in Estrada et al. [17] and Duncan et al. [28]. All qPCR reactions for each sample were performed in triplicate with the LightCycler 480 Real-Time PCR System (Roche Diagnostics). Beta-2-microglobulin (*B2M*) was chosen as the reference gene because of its minimum coefficient of variation between samples.

Validation of the 11 genes with positive results in the expression analysis described previously was performed with new assays designed using the online ProbeFinder software (Roche Diagnostics). Glyceraldehyde-3-phosphate dehydrogenase (*GAPDH*) and 18 S ribosomal (*18* *S*) were tested as possible reference genes, and *GAPDH* was selected. For this validation step, samples from three additional control individuals were included in the analysis. Again, all qPCR reactions for each sample were performed in triplicate with the LightCycler 480 Real-Time PCR System (Roche Diagnostics).

### Statistical Analysis

Linear regression was analysed by first testing the most important assumptions (normality and homoscedasticity). Calculations were performed with SPSS v11.5 (SPSS, Chicago, IL, USA). To test skewness (or asymmetry of a distribution) of the genetic risk distributions of the HBM cases and the BARCOS controls, we used the robust medcouple (MC) measure, with left and right tail weight measures (LMC and RMC), and constructed the MC-LR confidence interval [29].

## Results

### HBM Prevalence in the BARCOS Cohort and Features of the HBM Cases

In total, 1600 DXA scans were analysed across the BARCOS cohort. Those cases in which the sum Z-score was equal to or greater than four were considered HBM cases and further analysed. Pathologic phenotypes were ruled out based on a more in-depth examination of the medical history, a physical examination and a radiologic study. In the BARCOS cohort, 10 cases (0.63% of individuals) fulfilled this HBM criterion. Six additional HBM cases were recruited elsewhere (see Materials and Methods). Z-score values, age and cohort for all HBM cases are listed in Table 1.

### Search for Mutations in *LRP5* and *DKK1*

**Figure 1:**
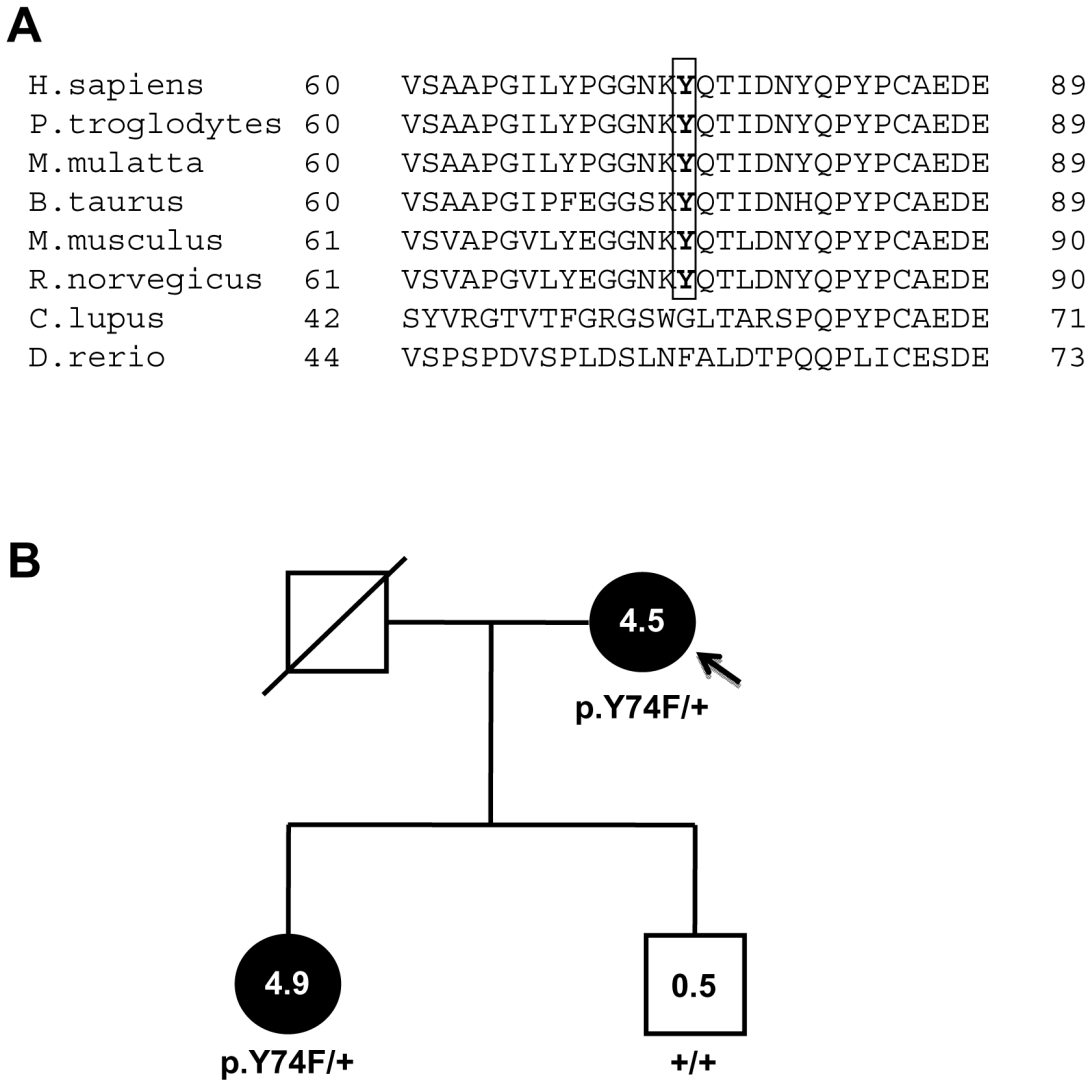
Mutation p.Y74F of *DKK1* may be responsible for high bone mass in family HBM15. (**A**) Alignment of a partial human *DKK1* sequence with those of several vertebrates. The tyrosine-74 residue is boxed. (**B**) Pedigree of case HBM15 (arrow): filled symbols indicate a high bone mass phenotype. Numbers inside symbols are sum Z-score values.

Exons 2 to 4 of *LRP5*, which encode the first beta-propeller of the protein, and in which HBM mutations have previously been described, were sequenced in the 13 HBM cases with available DNA sample. Next, exons 9 to 16 were analysed, since they encode beta-propellers 3 and 4, which have been described as binding regions for the LRP5 inhibitor DKK1. No novel or previously described causing mutations were found in any of these exons. The missense *LRP5* polymorphisms p.V667M (rs4988321) and p.A1330V (rs3736228), associated with BMD in GWA studies, together with other silent exonic variants found in the HBM individuals, are shown in Table 2. Their frequencies in HBM cases were similar to those found in the general population (dbSNP; http://www.ncbi.nlm.nih.gov/SNP/). Regarding *DKK1*, the four exons and flanking regions were amplified and sequenced in the 13 HBM cases. One previously undescribed heterozygous missense change (p.Y74F) and an exonic silent polymorphism were found in different individuals (Table 2). The p.Y74F was not present in the 1000 genomes database, while the tyrosine 74 and adjacent residues were found to be conserved in the Dkk1 sequences of primates, rodents and cows, but not in *C. lupus* or *D. rerio* (Fig. 1A). PolyPhen-2 (HumDiv) and SIFT scores for this missense change were as follows: 0.48 (possibly damaging) and 0.38 (tolerated), respectively. Examination of the offspring of case HBM15 revealed a cosegregation of this mutation with the HBM phenotype (Fig. 1B). The daughter was found to be an HBM case (sum Z-score: 4.9) and was heterozygous for the mutation, while her brother had a normal sum Z-score value (0.5) and did not carry this DNA change.

### Analysis of 55 Bone Mineral Density *Loci*

**Figure 2:**
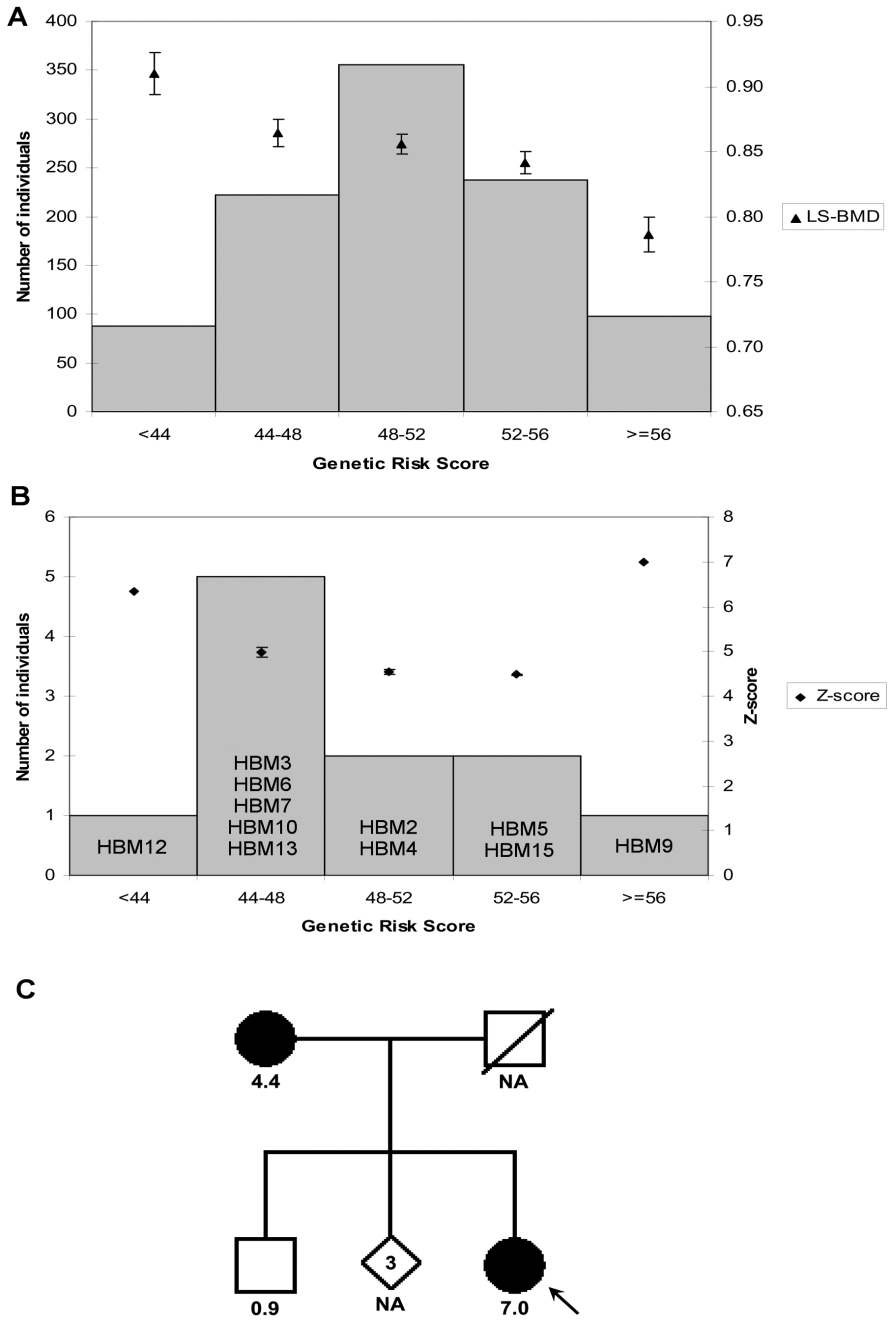
Distribution of genetic risk scores in BARCOS and in the HBM group. Distributions of genetic risk scores among 1001 BARCOS individuals (**A**) and 11 HBM probands **(B**), and their relationships with BMD or Z-score values, are shown. Histograms describe counts of individuals in each genetic score category (left axis scale); (**A**) From left to right, exact numbers of individuals in each bin are: 88, 222, 355, 238 and 98. Triangles (right axis scale) represent LS-BMD means and vertical bars depict their standard errors; (**B**) Diamonds represent mean Z-score values. (**C**) Pedigree of family HBM9. Arrow indicates the proband HBM9; filled symbols represent presence of the HBM phenotype; numbers below symbols denote sum Z-scores; NA: not available.

Figure 2A shows the distribution of the BARCOS individuals into five different osteoporosis risk score bins (bars) and the mean LS-BMD value for each bin (triangles). Risk scores were derived from the number and effect of the BMD-associated SNPs described in Estrada et al. [17] (see Methods). As expected, a decrease in BMD values was observed as the genetic risk score increased (Pearson correlation coefficient  = −0.972, p = 0.0057, r^2^ = 0.94). Figure 2B shows a similar graph for the 11 HBM individuals for which genotyping was successful (two of the HBM cases -HBM14 and HBM16- had to be discarded because of sub-optimal genotyping results). The frequency distribution shows a shift towards the lower genetic risk score bins. Again, BMD values (measured as Z-scores) decreased as genetic risk scores increased, with the exception of one individual (HBM9), who presented the maximum BMD value (Z-score  = 7) and the largest genetic risk score. Interestingly, the HBM phenotype in the family of HBM9 seems to segregate as a discrete trait: the mother is also a HBM individual (sum Z-score  = 4.4), while her eldest brother is not (Fig. 2C). A correlation analysis between risk scores and Z-scores of the 11 HBM individuals was not possible due to the small sample size, since linear regression assumptions were not fulfilled. To compare the two distributions their skewness was analyzed. MC-LR 95% confidence intervals for skewness [(−0.193, 0.116) for Fig 2A and (−1.528, 0.740) for Fig 2B] pointed to a loss of symmetry for Fig 2B. However the intervals did overlap, due to the limited sample size of the HBM group. Thus, significant differences between the two groups could not be formally demonstrated.

### Expression Analysis of 88 Bone-development and/or Wnt-pathway Genes

**Figure 3:**
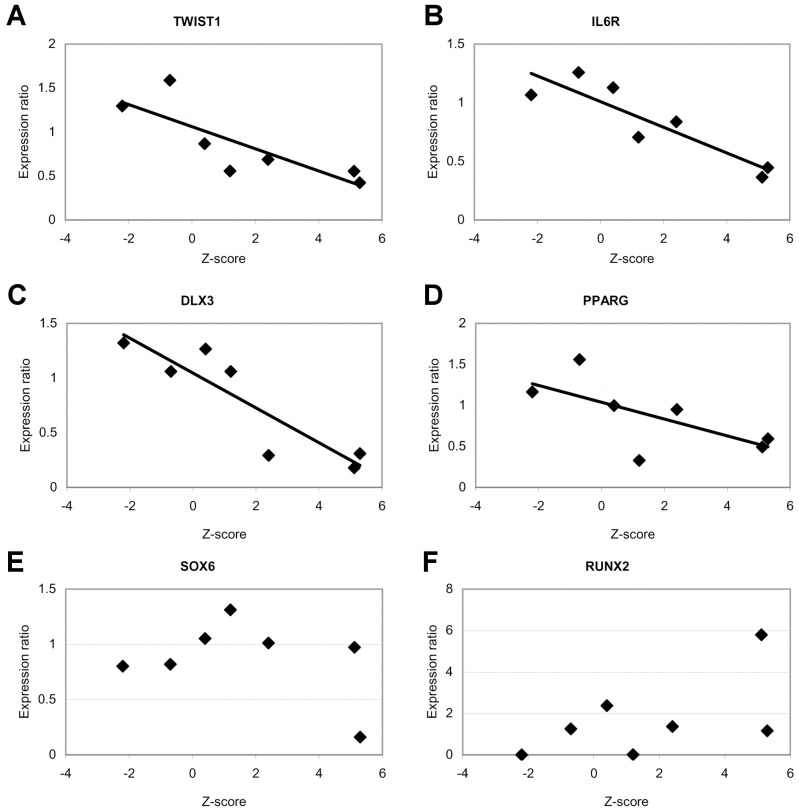
Analysis of mRNA levels of several candidate genes in relation to BMD levels. (**A**–**D**) Trend of correlation between Z-score values and gene expression levels of **(A)** *TWIST1*, (**B**) *IL6R*, (**C**) *DLX3* and (**D**) *PPARG*. (**E**) One of the HBM samples presented an expression level of *SOX6* 5-fold decreased in relation to the mean of five control individuals. (**G**) The other HBM sample presented an expression level of *RUNX2* that was 6-fold increased.

A transcriptomic analysis by qPCR was carried out in primary osteoblast samples from two HBM and two age- and gender-matched control individuals that were obtained after knee-replacement orthopaedic surgery. Because of the small sample size, the approach was only meant to be descriptive. In a first step, 11 out of 88 bone-development and/or Wnt-pathway genes were selected (Table 3) due to differences above or below 2-fold in mean expression level between the two HBM cases and the two control individuals. Subsequently, these 11 genes were re-analysed using samples from the two HBM and five control individuals (the initial 2 controls, whose Z scores were negative, and 3 new ones with Z-scores of 0.4, 1.2 and 2.4). Because the sum Z-scores of the control individuals were scattered across a wide range of values, expression levels were plotted against sum Z-scores and we observed that *TWIST*, *ILR6*, *DLX3* and *PPARG* displayed a trend of correlation (Figure 3,A–D). R^2^ values for the four genes were 0.676, 0.895, 0.807 and 0.461, respectively. However, due to the small sample size, linear regression assumptions were not fulfilled. Thus, no p-values are provided. We also observed that for *SOX6* and *RUNX2*, one of the HBM samples (but not the other) presented an expression level that was 5-fold decreased or increased, respectively, compared with controls (Fig. 3E and 3F, respectively).

**Table 3:** Eleven genes selected from the RealTime Custom Panel^1^.

Wnt-pathway genes	Bone biology genes		
*FZD3*	*BMP4*	*IL6R*	*SOX6*
*SOST*	*COL10A1*	*PPARG*	*SP7 (OSX)*
	*DLX3*	*RUNX2*	*TWIST1*

1 Those displaying at least a two-fold difference between the mean expression levels of HBM10 and HBM16 and the mean of the two controls with negative Z-scores.

## Discussion

In this study we established that the prevalence of HBM in a Spanish cohort of postmenopausal women is 0.63%. None of the HBM individuals had mutations in the relevant exons of the *LRP5* gene that could explain their phenotype. One individual had a rare missense change in *DKK1* (p.Y74F). The results of the analysis of 55 osteoporosis-predisposing SNPs pointed to an inverse correlation between risk alleles and BMD in this group of HBM women, with the exception of one case with the highest BMD value and the highest risk score. Finally, the results of an expression analysis in primary osteoblasts showed a negative trend between Z-scores and mRNA levels of *TWIST1, IL6R, DLX3*and *PPARG*.

There are few studies that describe the prevalence of HBM in the general population. In a recent one [15], the authors studied a UK DXA-scanned population in which the prevalence of HBM was 0.2% of individuals. The lower prevalence (0.2% versus our 0.6%) may be due to differences in the study design, including the definition of HBM, which was stricter in their study. All HBM-related *LRP5* mutations identified to date are located in the first beta-propeller of the protein [10], while the mutations that cause osteoporosis-pseudoglioma syndrome and exudative vitreoretinopathy are found all over the gene [30]. In our mutational analysis of exons coding for the first beta-propeller (and for the third and fourth, which have been described as binding regions for DKK1 [31]) no mutations were detected in the 13 HBM individuals analysed. Our results are in agreement with those published by Duncan et al. [32], who pointed out that <2% of their HBM cases were due to mutations on exons 2–4 and intron/exon boundaries of the *LRP5* gene, after analysing 98 patients.

We also analysed the *DKK1* gene under the hypothesis that loss-of-function mutations in this gene could have the same effect as gain-of–function mutations in *LRP5*. In this regard, it has been shown that bone mass was inversely proportional to Dkk1 levels in mice [33], and there are therapies under development based on *DKK1* inhibition to increase bone mass (reviewed in Ke et al. [34]). We found a missense change (p.Y74F) in heterozygosis in one HBM individual. We have gathered some evidence that supports a causative role for this mutation. The change affects a conserved residue and it is predicted to be possibly damaging. Additionally, it cosegregates with HBM in the nuclear pedigree of case HBM15. In humans, the only report on *DKK1* mutations is by Korvala et al. [35], who recently suggested that a mutation in *DKK1* may predispose individuals to primary osteoporosis. No mutations were found in the HGMD Professional 2012.4 database (released 29 March 2013), and a limited number of very rare missense changes (31) were found in the 1000 genomes database (released 13 December 2012); eleven of these are predicted to be deleterious by SIFT and probably damaging by PolyPhen. Whether these changes, or the p.Y74F described in this paper, are associated with a HBM phenotype remains an interesting open question, which will require functional analyses to be confirmed.

To test whether HBM could be explained by a polygenic additive effect of susceptibility loci, we chose to genotype the GWA-discovered BMD loci defined by Estrada et al. [17]. We were able to use the BARCOS cohort information for these same loci as a framework for comparison. We would expect HBM individuals to bear a high number of protective alleles or a low number of risk alleles. As seen in Figure 2, the distribution of genetic risk scores for the HBM cases shifted towards lower risk, with the mode bin set at 44–48 instead of 48–52 and lost symmetry. However, due to the limited number of HBM cases, statistical significance was not achieved and, thus, no significant differences between the small cohort of individuals with HBM and the BARCOS cohort could be demonstrated.

Additionally, Z-score values decreased as genetic risk scores increased, suggesting that common variation is playing a role in determining HBM. However, we note that the only HBM individual falling into the highest risk score bin (HBM9) was the one with the highest Z-score and such a contradictory fact might indicate the existence of a highly penetrant and probably rare protective allele counterbalancing the additive effect of the risk alleles. The HBM phenotype of this individual might be a Mendelian trait due to an as-yet unidentified gene. Altogether, these results are consistent with the coexistence of both polygenic and Mendelian cases of HBM, which would then be a heterogeneous trait.

To gain further insight into specific genes that might be involved in HBM, we undertook a descriptive transcriptomic study of two HBM cases for which we had access to primary osteoblast cultures. When expression levels were compared between the HBM cases and five controls, it was interesting to find four genes that presented mRNA levels displaying a negative trend with BMD (*TWIST1, IL6R, DLX3* and *PPARG*). Two other genes had one of the two HBM samples with outlier mRNA levels: *RUNX2* was 6-fold elevated in one HBM sample, while *SOX6* was 5-fold reduced in the other HBM sample. In spite of the evident low sample size, it is tempting to speculate that these genes (among others) may play a role in HBM, acting in an additive way.

The limitations of this study include the small sample size of HBM cases, which precluded reaching significance. Replication in other cohorts will be necessary to confirm some of our results. This is particularly important in the expression analysis, where the number of analysed individuals was modest because of the great difficulty in finding primary human osteoblasts. It may be argued that the fact that the donors suffered from arthritis is a confounding factor. However, all donors were affected with this condition regardless of their BMD and it might be assumed that the effect of artritis would be similar in all of the samples. To minimize the impact of arthritis, caution was taken to obtain the osteoblasts from locations far away from the lesion. However, a systemic effect of arthritis cannot be totally ruled out.

In conclusion, to our knowledge, this genomic and transcriptomic analysis of HBM is the first report of its kind. By combining both strategies, it was possible to gain a deeper insight into the genetic makeup of HBM. It includes suggestive evidence of genetic heterogeneity based on the observation of additive effects of several genes, on one hand, and monogenic cases not caused by *LRP5*, on the other. *DKK1*, possibly responsible for one of these monogenic cases, would be a novel HBM gene. Future studies in enlarged cohorts may confirm the relevance of the genes described here, some of which might be therapeutic targets for osteoporosis.

## Supporting Information

Table S1 **Primers and PCR conditions for the amplification of selected** ***LRP5*** **and** ***DKK1*** **exons.**
(DOC)

Table S2 **List of the 55 SNPs genotyped in the HBM cases.**
(DOC)
