# Supplementary material for: Genetic Analysis of High Bone Mass Cases from the BARCOS Cohort of Spanish Postmenopausal Women
Source: PLoS One. 2014 Apr 15;9(4):e94607. doi: 10.1371/journal.pone.0094607 (PMC3988071; doi:10.1371/journal.pone.0094607)
Supplement: Table S1 — Primers and PCR conditions for the amplification of selected LRP5 and DKK1 exons. (DOC) [file pone.0094607.s001.doc]

Table S1. Primers and PCR conditions for the amplification of selected *LRP5* and *DKK1* exons
