# Supplementary material for: Genetic Analysis of High Bone Mass Cases from the BARCOS Cohort of Spanish Postmenopausal Women
Source: PLoS One. 2014 Apr 15;9(4):e94607. doi: 10.1371/journal.pone.0094607 (PMC3988071; doi:10.1371/journal.pone.0094607)
Supplement: Table S2 — List of the 55 SNPs genotyped in the HBM cases. (DOC) [file pone.0094607.s002.doc]

**Table S2.** List of the 55 SNPs genotyped in the HBM cases

rs163879

rs227584

rs344081

rs430727

rs479336

rs884205

rs1053051

rs1026364

rs1286083

rs1346004

rs1366594

rs1373004

rs1566045

rs1864325

rs1878526

rs2016266

rs2062377

rs2887571

rs3736228

rs3755955

rs3790160

rs3801387

rs3905706*

rs4233949

rs4727338

rs4790881

rs4796995

rs4869742

rs4985155

rs6426749

rs6532023

rs6959212

rs7017914

rs7071206

rs7084921

rs7108738

rs7217932

rs7584262

rs7812088

rs7851693

rs7932354

rs7953528

rs9466056

rs9533090

rs9921222

rs10048146

rs10416218

rs10835187

rs11623869

rs11755164

rs12407028

rs12821008

rs13204965

rs13336428

rs17040773

* This SNP was eliminated from the analyses due to conflicting results.
